# Visual and refractive outcomes of new intraocular lens implantation after cataract surgery

**DOI:** 10.1038/s41598-022-14315-6

**Published:** 2022-08-18

**Authors:** Bhupesh Singh, Sourabh Sharma, Neha Bharti, Dharitri Samantrey, Dadan J. Paandey, Sudhank Bharti

**Affiliations:** Bharti Eye Center and Foundation, New Delhi, India

**Keywords:** Health care, Therapeutics

## Abstract

To report the visual and refractive outcomes of new aspheric hydrophobic acrylic monofocal intraocular lens (IOL). Retrospective case series. This study included eyes of patients who underwent routine cataract surgery for uncomplicated age-related cataract with implantation of a Aktis SP (NS-60YG; Nidek Co. Ltd., Japan) IOL and attended regular follow ups at 1 week, 1 month, 3 months, and 12 months. At each post-operative visit, ophthalmological evaluation included measurement Uncorrected (UCVA) and Best corrected visual acuity (BCVA), contrast sensitivity, posterior capsular opacification (PCO), optical aberrations, analysis of point spread function (PSF) and modulation transfer function (MTF). The study included 2102 eyes of 1358 patients aged 45 to 75 years (mean age 62.6 years ± 5.6 SD). The mean preoperative BCVA was 0.56 ± 0.26 logMAR. At 1 year follow up, the mean postoperative UCVA and BCVA were 0.11 ± 0.09 and 0.02 ± 0.03 logMAR, respectively. At the end of 6 months, around 1487 (93%) eyes had BCVA of 20/20 and better than 20/30 in 100% of the eyes. Mild posterior capsule opacification (PCO) was observed in 56 patients, but none required Nd YAG laser capsulotomy. There was reduction in ocular spherical aberration and Higher order aberrations (HOAs) as compared to pre operative. This explains better contrast sensitivity obtained by MTF and PSF values. The study shows that the Aktis SP IOL is safe, effective, and stable lens that could be inserted through 2.2 mm incision with satisfactory visual and refractive outcomes, even in late post-operative period.

## Introduction

A paradigm shift has been seen in the outcomes of cataract surgery over years^1^. The credit not only goes to improved cataract surgery techniques and technologies like microincision phacoemulsification and femtosecond laser assisted cataract surgery, but also to marked improvement in intraocular lenses (IOLs)^2–5^. The first IOL was implanted in 1949 by Sir Harold Ridley. Since then IOLs have undergone revolutionary changes. Monofocal IOLs, which restore unaided visual acuity for far vision, are currently the most commonly used^6^. However, these patients need reading glasses to compensate for the loss of ability to see near distances. In the last one-decade, we have witnessed a variety of newer concept IOLs like multifocal IOLs, trifocal IOLs, extended depth of focus lenses^7–10^. Diffractive and refractive designs IOLs are also used. Newer technologies like enlighten optical technology and convolution processes have been developed to reduce the light loss and sharper vision in trifocal IOLs^11^. Most of these advances in IOL technology aim at providing good vision at all distances that is near, intermediate and far. All these lenses have some advantages and also have their own set of problems. The problem of glare and haloes makes these multifocal IOLs not suitable for many patients^7–10^. The other limitation of these IOLs is that they are not suitable in patients with diabetic retinopathy, glaucoma and poor ocular surface. The steep cost of these premium IOLs must also be taken into consideration. These limitations make trifocal lenses not so commonly used in comparison to monofocal IOLs. In monofocals, the neuroadaptation is better and patients have lower rate of visual disturbances^12^. In Indian scenario, monofocal IOLs still represent the most used ones in the current surgical practice. The previous generation of monofocal IOLs have been reported to have higher rates of posterior chamber opacification (PCO)^13^. Newer monofocal IOL design, larger optic zones and square edged optic design has significantly improved the outcomes of cataract surgery and reduced the PCO^14–16^. Nowadays, the aim of IOL implantation is not only to attain post-operative emmetropia but also to improve factors like optical quality and contrast sensitivity. An in vitro experimental study of NS-60YG (NIDEK) IOL demonstrated that this IOL showed fewer glistenings and long-term stability with no signs of deterioration due to ageing^17^, but clinical evidence is not yet available. The current study aims to evaluate the visual and refractive outcomes of newer aspheric monofocal IOL Aktis SP (NS-60YG; Nidek Co. Ltd., Japan).

## Materials and methods

This was a retrospective non-comparative chart review including patients who were scheduled to undergo routine cataract surgery with implantation of Aktis SP monofocal IOL implantation between July 2017 and October 2019. All the study procedures and surgeries were performed at a single centre (Bharti eye hospital and foundation). The study conduct adhered to the tenets of Declaration of Helsinki, and the Bharti eye foundation ethics board committee reviewed and approved the study. The patients were selected from the consecutive cases from the clinic population who were eligible for routine phacoemulsification cataract surgery and IOL implantation. Subjects were excluded from the study if they were unable to comprehend written or spoken language or unable to give informed consent, if they had any ocular disease other than cataract and previous ocular surgery or inflammation. Other exclusion criteria include presence of amblyopia, previous refractive surgery, significant ocular surface disease, glaucoma, existence of corneal, retina or optic nerve disease, axial lengths of < 22 or > 26 mm, astigmatism of 1.00 D or higher. Written informed consent to participate in the study was obtained from all subjects^7^.

### Intraocular lens

**Figure 1:**
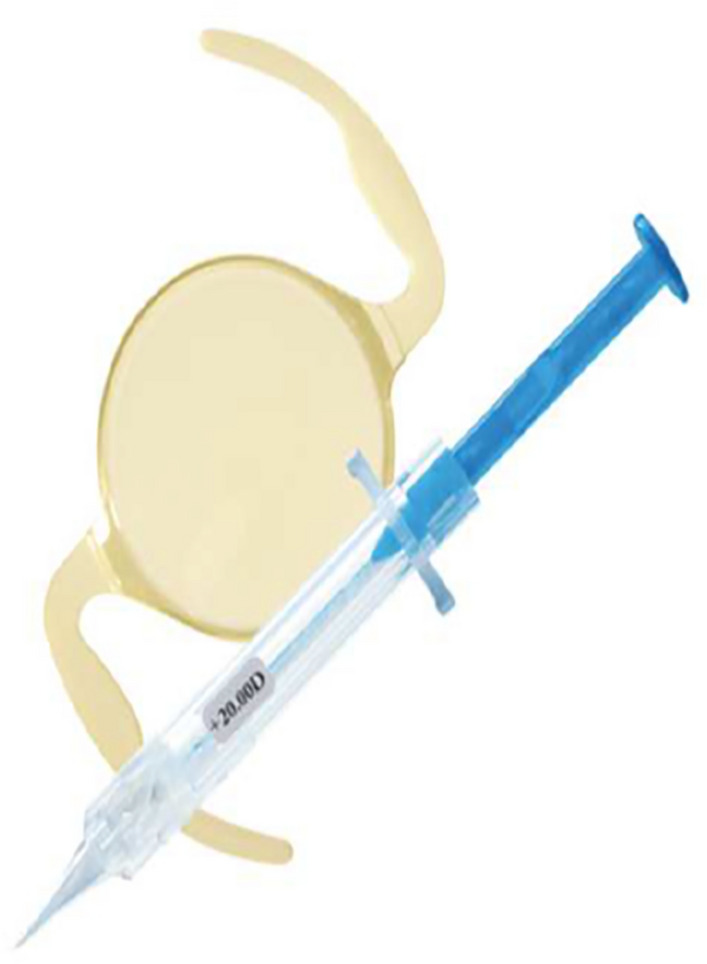
Aktis SP NS-60YG IOL with pre-loaded injector.

The study IOL was the Aktis SP (NS-60YG; Nidek Co. Ltd., Japan), which is a one-piece monofocal hydrophobic acrylic IOLs with modified C-loop haptics, 6 mm optic diameter and a total diameter of 13 mm (Fig. 1). The IOL is made of copolymer of n-butyl acrylate, n-butyl methacrylate and phenoxyethyl acrylate with asperitic optic side surface. The lens is injected into the eye using the preloaded IOL injection system which enables 2.2 mm in-the-bag implantation. It has a continuous 360° square edge profile which intended to help provide a barrier against cell migration. The 90° haptic design enlarges the contact area with the capsular bag resulting in a stable, well-centered IOL implantation while the asperitic optic side surface reduces intraocular reflection and optic edge glare. The IOL has a blast finishing on the haptic surface which reduces adherence to the optic surface during lens folding and improves the visibility of the haptic when the lens is loaded into the cartridge^18^.

### Patient assessment

All subjects underwent a standardized pre-operative ophthalmic examination including measurements of uncorrected visual acuity (UCVA) and best corrected visual acuity (BCVA) for distance, manifest refraction, slit-lamp examination and biomicroscopy, Goldmann applanation tonometry, optical biometry, keratometry, endothelial cell count (CEM 530, Nidek Co. Ltd., Japan) and retinal evaluation under pupil dilation. The patients were evaluated post-operatively at 1 week, 1 month, 3 months, 6 months and 12 months after surgery. The main outcome measure was distance visual acuity. The CDVA was evaluated at all postoperative visits. Distance vision was measured with logMAR notation using the Early Treatment Diabetic Retinopathy Study (ETDRS) charts (ETDRS; Vector Vision, Ltd, Greenville, OH, USA) at 4 m. The visual acuity testing was done under photopic conditions (85 candelas [cd]/m2) and at 100% contrast. Other outcome measures were optical performance of IOL. The optical quality of IOL was assessed by modulation transfer function (MTF) and the point-spread function (PSF), using a corneal analyzer system (OPD-Scan III, Nidek Co., Ltd.) The software obtains PSF, expressed as the Strehl ratio, which is the ratio between the intensity of the real PSF and the intensity of the diffraction-limited PSF. The assessment was done at 3.0 mm and 5.0 mm pupil diameters (PD). The PSF describes the quality response of an imaging system and is expressed by the Strehl ratio, with 1 indicating a perfect optical system. The mean MTF values were calculated in a logarithmic plot and log base 10 contrast sensitivity values were used to construct a graph for each spatial frequency tested. Patients also underwent wavefront aberrometry (OPD scan III, NIDEK, Japan) for evaluation of wavefront total spherical aberration and higher order aberrations (HOAs). Optical biometry was done on AL scan (NIDEK, Japan). SRK/T formula was used to calculate the IOL power. Company-labeled A-constant was 119.6. All patients underwent uneventful phacoemulsification. Cataract surgery with implantation of Aktis SP hydrophobic acrylic monofocal IOL by our experienced surgeons. A standard sutureless microincision (2.2 mm) phacoemulsification technique was used. All surgeries were performed on Infiniti Vision system (Alcon, USA). The anterior capsulorhexis of approximately 5.0 mm diameter was created and the IOL was implanted in the capsular bag with manufacturer’s recommended IOL loading and injection technique. Postoperatively, topical therapy included a combination of antibiotics and steroidal agents. The eyes were targeted for emmetropia.

### Statistical analysis

Statistical analyses were performed using SPSS statistics for Windows (IBM SPSS Statistics for Windows, Version 19.0., Armonk, NY). Normality was checked using the Kolmogorov–Smirnov test. Statistically significant differences were determined using the Student t test and paired-sample t test. Statistical significance was set at P < 0.05.

## Results

Total 2340 eyes of 1553 patient underwent cataract surgery with Aktis SP NS-60YG IOL implantation during the study period. Of these, 2102 eyes of 1358 patients were included in the study. 238 eyes were excluded from the review because of associated ocular pathology other than cataract. The most common factors that were responsible for exclusion of these eyes were diabetic retinopathy, glaucoma, Age related macular degeneration, amblyopia and severe dry eye. Four eyes of 4 patients required additional IOL repositioning in the post-operative period and were excluded from the study. All patients completed all follow-up examinations up to 12 months postoperatively. The patients were aged 45 to 75 years (mean age 62.6 years ± 5.6 SD). Patient characteristics and preoperative data are shown in Table 1.

**Table 1:** Patient characteristics and preoperative data.

Parameter	Value
Patient/eyes (n)	900/1598
**Age (years)**	
Mean ± SD	62.6 years ± 5.6 (range 45 to 75)
**Sex (n)**	
Male	340
Female	560
**Eye (n)**	
Right	856
Left	742
**Pre-op monocular CDVA (logMAR)**	
Mean ± SD	0.56 ± 0.26 (range 0.24 to 1.20)
**Spherical equivalent (D)**	
Mean ± SD	0.75 ± 1.06 (range -3.50 to 2.40)
**IOL power (D)**	
Mean ± SD	21 ± 2.8 (range 17–26.5)

**Figure 2:**
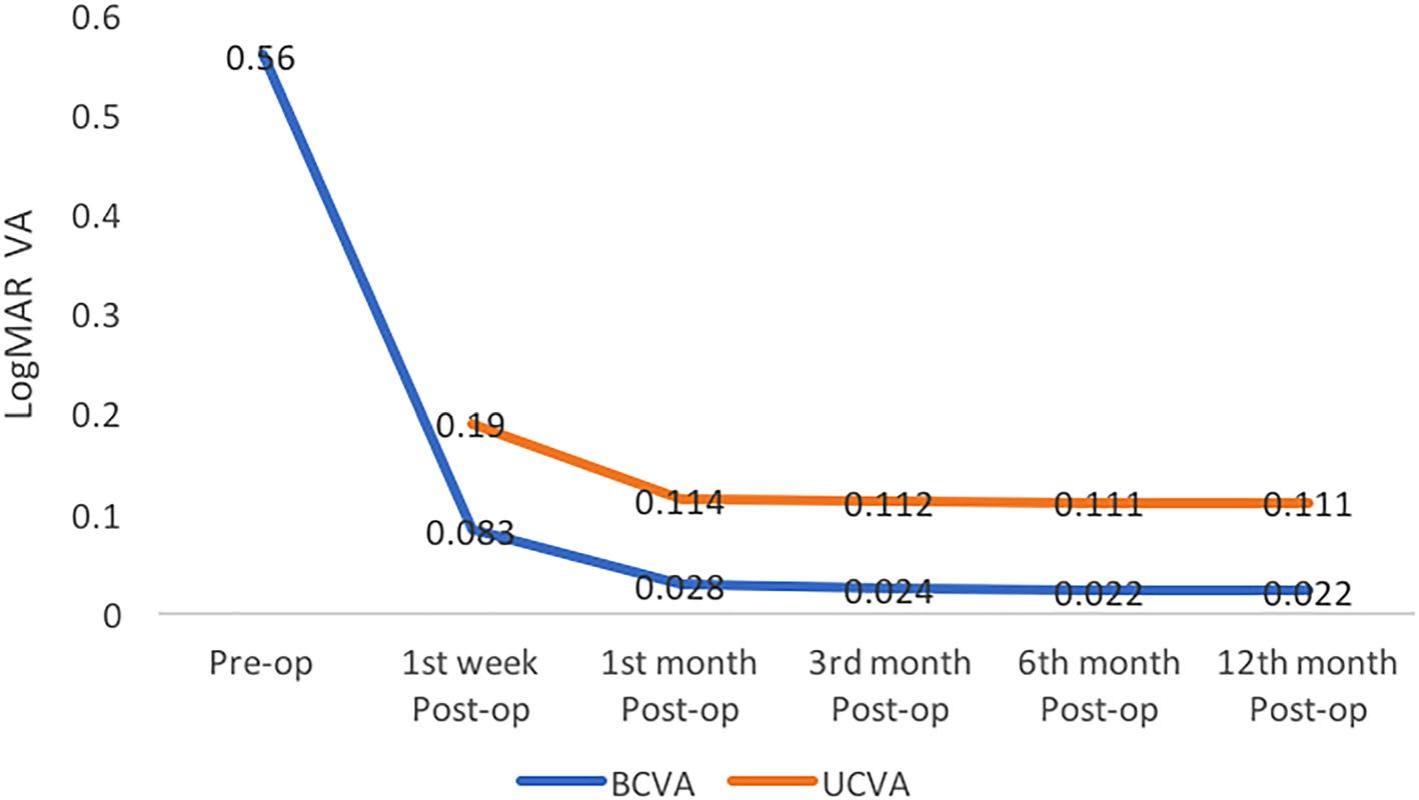
Mean uncorrected and best corrected visual acuity (UCVA and BCVA) over time at all post324 operative visits in LogMAR.

**Figure 3:**
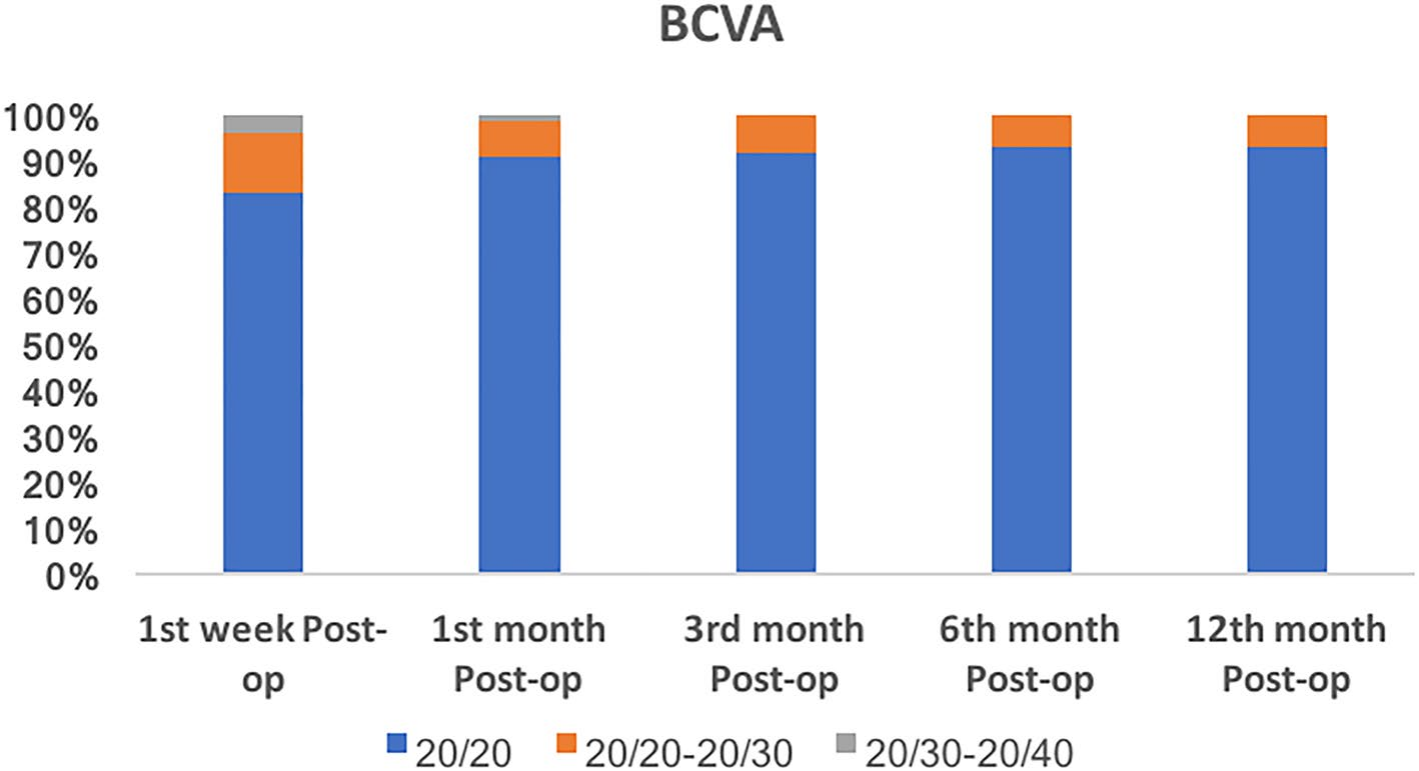
Time course of changes in the best corrected visual acuity (BCVA).

**Figure 4:**
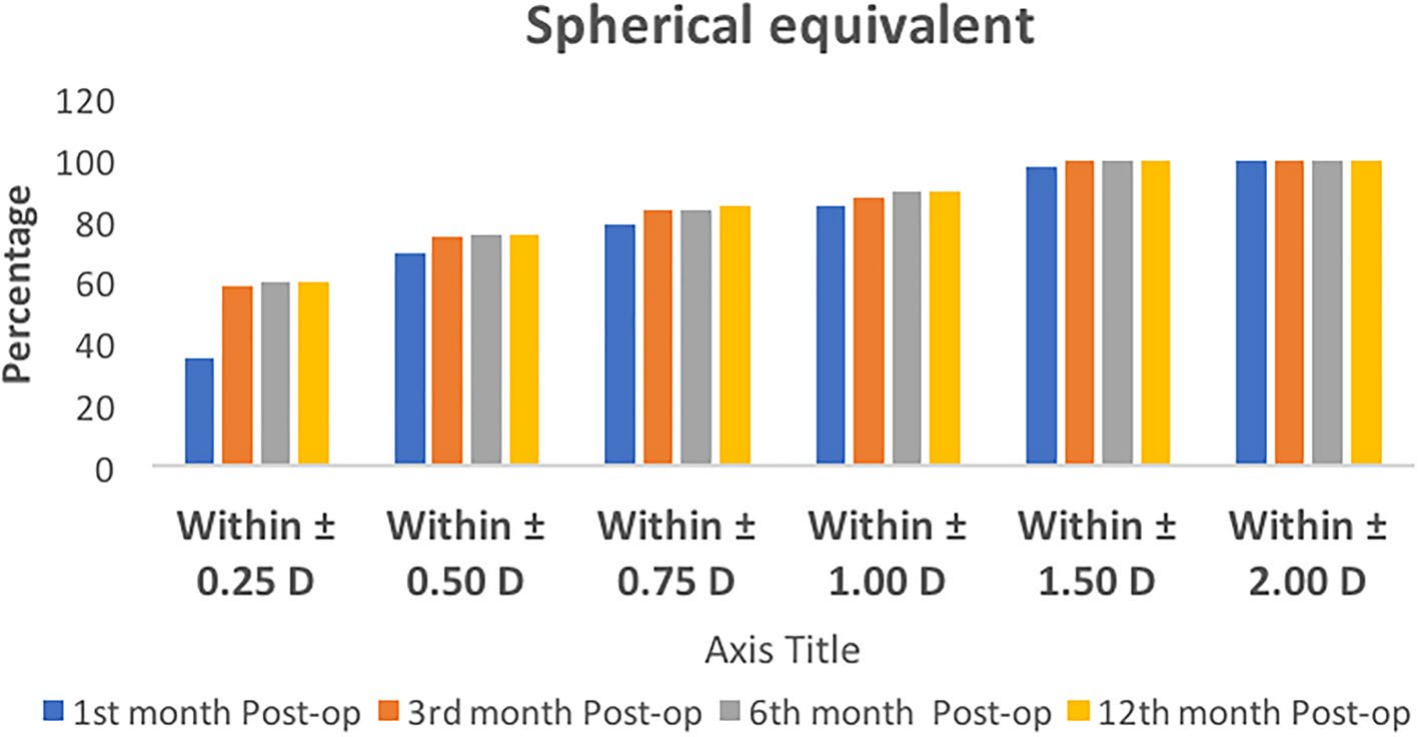
Cumulative bar graph of predictability for spherical equivalent at various post-operative visits.

The mean UCVA and BCVA over time at all post-operative visits are shown in Fig. 2. On the first postoperative day, a CDVA of 20/20 was achieved in 1325 (83%) eyes. At the end of 6 months, around 1487 (93%) eyes had BCVA was 20/20 and better than 20/30 in 100% of the patients (Fig. 3). The post-operative mean SE and the mean refractive astigmatism were − 0.25 ± 0.39 D and − 0.46 ± 0.30D, respectively, at 1 year follow up. During the entire follow-up period, there were no significant fluctuations in the postoperative refraction. The spherical equivalent was within ± 0.5 D in 76% and ± 1.0 D in 90% eyes at 12 months postoperatively (Fig. 4). There was a mean reduction of 0.87% endothelial cell loss at 12 months post-operative period. The mean corneal endothelial cell density preoperatively and at 12 months postoperatively was 2756 ± 176 per mm^2^ and 2516 ± 207 per mm^2^ 169, respectively. At the end of 12 months, none of the cases had required laser capsulotomy.

Though mild posterior capsule opacification was observed in 56 patients on subjective evaluation, but the visual acuity was not affected. The mean BCVA among these 56 eyes were 0.029 ± 0.02 logMAR. In our cohort, 1852 eyes have completed 3 years follow up and 148 of these eyes showed mild posterior capsular opacification on subjective evaluation but there was no decrease in visual acuity The mean BCVA among these 1852 eyes were 0.023 ± 0.02 logMAR.

**Figure 5:**
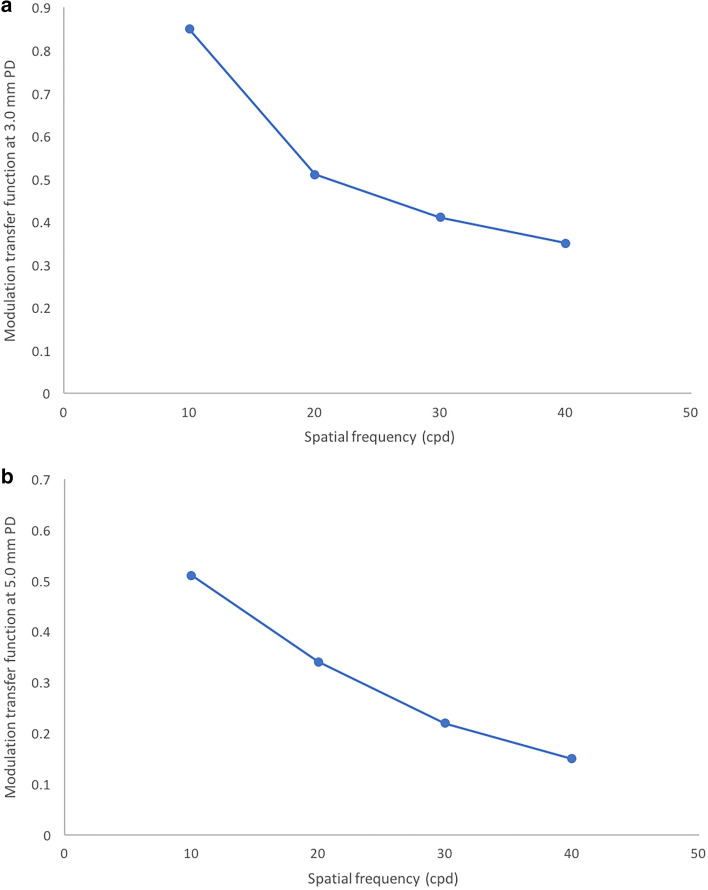
(**a**) Modulation transfer function curve with a 3.0 mm pupil diameter at different spatial frequencies at 12 months post-operative (cpd—cycles per degree). (**b**) Modulation transfer function curve with a 5.0 mm pupil diameter at different spatial frequencies at 12 months post-operative (cpd—cycles per degree).

Figure 5a,b shows the MTF function curves at different spatial frequencies with a 3.0 mm and 5.0 mm pupil diameter at 12 months post-operative respectively. The Strehl ratio values with a 3.0 mm and 5.0 mm PD at 1 year post-operative follow-up were 0.84 and 0.79 respectively. The pre and post-operative values of spherical aberration and HOA for 3 mm pupil and 5 mm is shown in Table 2. The postoperative wavefront analyses, including the mean HOA values and spherical aberration at 1 year visits for the two groups were found to be statistically significant than baseline. None of the patients complained about photopsia while glistenings and surface light scattering were not observed in any eye.

**Table 2:** Analysis of Spherical aberration and Higher order aberrations at 3 and 5 mm pupil size.

	Baseline	1 month post-op	3 month post-op	6 month post-op	12 month post-op	P value
Spherical aberration	0.3	0.04	0.034	0.032	0.032	< 0.01
Higher order aberration (3 mm pupil)	0.65	0.40	0.44	0.45	0.47	< 0.05
Higher order aberration (5 mm pupil)	0.94	0.62	0.66	0.68	0.70	< 0.05

*P value for difference between preoperative and 6-month postoperative values.

During the entire follow-up period, there were no other significant intraoperative or postoperative complications such as intraocular pressure spikes, IOL decentration, tilting or dislocation, prolonged corneal edema or inflammations or any infective events. There was no secondary surgical intervention in the study group.

## Discussion

In this study, we analysed the visual outcomes and clinical performance of a hydrophobic acrylic monofocal IOL-NS-60YG. We found that this IOL was safe and effective with excellent postoperative vision, thereby providing satisfactory visual and refractive outcomes even in late postoperative period.

To our knowledge, this is first study to report the clinical outcomes in a cohort that had implantation of Aktis SP, NS-60YG IOL. Our findings showed that there was improvement in distance vision from 0.56 ± 0.12 to 0.22 ± 0.14 logMAR at 1 year post-op. The BCVA of the 637 eyes who were followed uptil 2 years was 0.24 ± 0.34 logMAR. These results are in agreement with findings obtained in previous studies with different IOL models^14,16,19–21^. We observed the post-operative mean SE to be -0.25 ± 0.39 D at 1 year follow up. Similar outcome has been observed in previous studies with different IOLs^14,16,24^. The mean refractive astigmatism of 0.25 ± 0.39 found in our study at 1 year follow up was too comparable to previous studies using monofocal IOLs^14,22^.

Technological advancements have brought about the use of acrylic made soft foldable inert materials which resulted in the development of aspheric IOLs to compensate for the corneal aberrations. Obtaining a satisfying refraction after cataract surgery is no longer the only goal of these new generation IOLs, but to also enhance the visual performance by improvement in contrast sensitivity function. Our study showed that the contrast sensitivity in terms of MTF and Strehl values achieved near normal values postoperatively, which has been previously documented in other studies with monofcal IOLs as well^23^.

In our study, we also evaluated spherical aberration and HOAs at 5 and 6 mm pupil size. The HOAs reflect the optical quality of the human eye after correction for sphere and cylinder. Data in our study showed that the wavefront analysis at all post-operative visits were found to be statistically significant than baseline, and implantation of an aspheric IOL leads to reduction in HOAs. As compared to previous studies, we found a higher amount of spherical aberration compared with that analyzed other types of aspheric IOLs, which have residual negative spherical aberration^24^.

The optics quality of the IOL was better with small pupils due to low aberration effect. This was seen in the graphs in which the curve was close to the diffraction curve, similar to what was described in previous studies^25^. One of the most significant complications of cataract surgery is PCO. In our study, 56 eyes (2.6%) had mild PCO at 1 year postoperatively, with none of the cases had requiring laser capsulotomy. The visual acuity of these 56 eyes were 0.029 ± 0.02 logMAR. Even among the 1852 eyes completing 2 years follow up, 148 of these eyes (7.99%) showed mild PCO on subjective evaluation but there was no decrease in visual acuity that could have necessitated laser capsulotomy. Similar rates have been observed in other studies^26–29^. Its known that IOLs with hydrophobic material and a sharp optic edge provide lower incidences of PCO^30–33^. The IOL in our study also had a 360° square edge, which protects against migration of the lens epithelial cells into the back of the optic and the haptic junction, minimizing the risk of PCO. There is double-polymerization of monomers in the IOL, which decreases voids in the IOL structure resulting in a stable polymer. This unique structural architecture inhibits water components from coalescing and reduces the chances of glistening and whitening and gives the IOL long-term stability. An experimental study study of NS-60YG IOL demonstrated that this IOL showed fewer glistenings and long-term stability with no signs of deterioration due to ageing. The results of that study evaluated the stability of IOL material simulating 20 years of ageing after implantation in an eye^17^.

To the best of our knowledge, this is a first manuscript which evaluates visual and refractive outcome of this hydrophobic aspheric monofocal IOL. The effectiveness of an IOL can be estimated by the visual outcomes and performance over a long follow-up period and this study has showed that Aktis SP NS-60YG IOL has good visual and optical quality even in the postoperative period. Significant contrast sensitivity enhancement and reduced spherical aberration was also seen.

## Data Availability

The datasets used and/or analysed during the current study available from the corresponding author on reasonable request.
